# Parental Perceptions of Child's Medical Care and Neighborhood and Child's Behavioral Risk Factors for Obesity in U.S. Children by Body Mass Index Classification

**DOI:** 10.1155/2019/3737194

**Published:** 2019-01-03

**Authors:** Joan A. Vaccaro, Gustavo G. Zarini, Fatma G. Huffman

**Affiliations:** Florida International University, Robert R. Stempel College of Public Health and Social Work, Department of Dietetics and Nutrition, 11200 SW, 8th ST, AHC-5 Room 324, Miami, FL 33199, USA

## Abstract

Environmental factors, preventive medical care, and behaviors play a role in childhood obesity. This study used the National Survey of Children's Health, 2011-2012, for 42,828 children, ages 10–17 years. Greater percent of children in the overweight/obese category performed no moderate-to-vigorous physical activity: 11.9 (10.6, 13.3) as compared to children in the underweight/normal weight category: 9.7 (8.9 10.6). No moderate-to-vigorous physical activity was associated with no preventive medical care, inadequate or no health care, parents reporting higher percent of no parks or playgrounds, and unsafe and unsupportive neighborhoods. Odds ratios of overweight/obesity were higher for males [OR = 2.06 (1.64, 2.60)], Hispanics [OR = 1.49 (1.17, 1.90)], non-Hispanic Black females [OR = 1.59 (1.20, 2.08)], younger females [OR_10–12 yrs._ = 1.35 (1.03, 1.79) and OR_13–15 yrs._ = 1.4. (1.06, 1.89) vs. OR = 1.00_16-17 yrs._], children with high television viewing [OR_0-1 hr./day_ = 0.72 (0.61, 0.86); OR_>1 to <4 hrs./day_ 0.84 (0.72, 0.99) = vs. OR = 1.00_≥ 4 hrs./day_,] and lower categories of physical activity [OR _0 days/wk._ = 1.38 (1.13, 1.62); OR_1–3 days/wk._ = 1.14 (1.22, 1.62) vs. OR_7 days/wk_. = 1.00], higher poverty, smoke exposure, and parental perception of their neighborhood as unsupportive. Promoting preventive medical care and neighborhood cooperation may have potential to lower childhood obesity.

## 1. Introduction

Childhood obesity is a public health issue. Children who are obese have more medical problems and cardiovascular risk factors as compared to their peers of normal body weight [1] and have a greater chance to become obese as adults [2]. Multiple environmental and behavioral factors contribute to risk of childhood obesity unequally among race/ethnicity and age groups [3]. Health care and neighborhood adequacy disproportionately affect children from minority and poor households. Pediatric preventive health care is considered key in obesity prevention for children [4, 5]. Childhood obesity experts advocated for obesity prevention training for physicians and other healthcare professionals [6]. Pediatricians can help prevent childhood obesity by preventive screening and discussions of body weight at primary care examinations [7]. Primary care childhood obesity interventions effective in weight reduction applied multiple behavioral strategies which targeted sedentary behavior, physical activity, and dietary behavior [8].

Key determinants of childhood obesity include neighborhood conditions, such as safety and greenspace [9–11]; family practices, for example, mealtime [12, 13]; degree of sedentary versus physical activity [14, 15]; and sleeping patterns [16, 17]. There is a gap in the literature as to how these determinants vary by gender and race/ethnicity. Certain social and behavioral determinants of childhood obesity were examined by Singh et al. [18] using the 2003 National Survey of Children's Health (NSCH); albeit, our study adds medical preventive care and health insurance adequacy, additional neighborhood characteristics, and family behavior using the 2011-2012 NSCH. This new survey includes both landline and cell phone participants. The aims of this study were (1) to determine the percent associations among adequate preventive care and adequate health care, with sociodemographics for U.S. children aged 10–17 years; (2) to measure the child's odds of being overweight or obese with parent's perceptions of their child's environment and parental report of their child's health behaviors, considering sex, race/ethnicity, and age and their interactions; (3) to test differences among race/ethnicity, neighborhood characteristics and health habits, and physical activity.

## 2. Methods

### 2.1. Study Design and Participants

This cross-sectional analysis was performed using data from the National Survey of Children's Health, 2011-2012 (NSCH) [19] which consists of 95,677 parental interviews of a single child (randomly-selected from households with more than one child) completed nationally with approximately 1,850 interviews collected per state (number of interviews per state range from 1,811 to 2,200) weighted to represent the population of noninstitutionalized children ages 0–17 nationally, and in each state. The present study was conducted in 42,828 children ages 10–17 years, for whom body mass index for age and sex (zBMI) in percentile categories was available. The unweighted sample included 4554 Hispanics (of any race); 4129 non-Hispanic Blacks (NHB); 29,892 non-Hispanic Whites (NHW); and 4253 from other or mixed races (Others).

### 2.2. Ethical Considerations and Data Availability

The data are publicly available [19], and the study was conducted by the Child and Adolescent Health Measurement Initiative (CAHMI) in accordance with the Declaration of Helsinki (1964) [20]. All interviews were conducted with the participants' understanding and consent, and the Ethical Committee has approved the interview protocol. The state and local integrated telephone survey (SLAITS) data collection is conducted under contract with NORC at the University of Chicago. Strict confidentiality and privacy regulations apply to all contract and federal project staff for all data. All respondents have agreed without coercion to participate in the survey and agreed to how the information would be used by the agency and for future analysis by the public.

### 2.3. Measures

The design and administration for the 2011-2012 NSCH were enhanced and differ somewhat from previous surveys: NSCH 2003 and 2007; key changes included the addition of “Amount of time spent using computers, cell phones, handheld video games, and other electronic devices” to the NSCH 2011-2012 [21] and surveying both landline and cell phone parents. The variables used for this study were constructed from the NSCH codebook for SPSS for complex sample analysis [22].

Overweight or obese was constructed from the variable body mass index for age and sex percentiles (zBMI): overweight 85% to <95% and obese ≥ 95% versus underweight/normal weight < 85%. The original variable of zBMI category was derived by the NSCH dataset based on the parental report of height and weight and using Center for Disease Control and Prevention's classification [23]. Preventive medical care was considered for responses of at least one visit per year and based on the following question, “During the past 12 months, how many times did [CHILD'S NAME] see a doctor, nurse, or other health-care provider for preventive medical care such as a physical exam or well-child checkup?” Insurance adequacy (meeting the child's needs and affordability) was compiled by NSCH as a health indicator and considered affirmative or negative, based on the parent's responses to questions concerning meeting the child's health needs with appropriate providers and reasonable out-of-pocket expenses. Age groups, created to test interactions with gender and race/ethnicity, were based on continuous data and grouped into preadolescence (10–12 years), early adolescence (13–15 years), and late adolescence (16-17 years). Physical activity was the parents' report of “times during the week that the child exercise, play a sport, or participate in physical activity for at least 20 minutes that made [him/her] sweat and breathe hard?” (moderate-to-vigorous level). There were insufficient numbers reporting zero times, so zero times was combined with 1–3 times. The variable was collapsed to three categories: participation for 0–3; 4-5; or 6-7 days per week. Adequate sleep was determined by parents' report of the number of days per week of their child getting enough sleep for the child's age. The variable sleep was recoded to 0–3; 4–6; and every night. Family meals (meals eaten together as a family at home or in a restaurant), on average, each week were coded as 0–3; 4-5; and 6-7 days. Children's major sedentary behaviors were based on two continuous variables that were recoded to the following three categories: less than 1 hour; 1 to less than 4 hours; and 4 or more hours per day. Television watching was based on parents' reports of the time their child spent watching television, videos, or playing video games. Electronic device use was constructed by the parental report of child's time spent using computer, cell phones, handheld video games, and other electronic devices, doing things other than schoolwork. Neighborhood characteristics included parent's perception as binary variables for the following: neighborhood safety (affirmative response to feeling safe in the community); availability of parks or playgrounds; and supportiveness of the neighborhood (NSCH derived from questions concerning cooperation and trust). Specifically, supportiveness of the neighborhood was converted by NSCH to a binary variable with agreement to at least three out of four statements (people in my neighborhood help each other out; we watch out for each other's children in this neighborhood; there are people I can count on in this neighborhood; and if my child were outside playing and got hurt or scared, there are adults nearby who I trust to help my child). Second- and third-hand smoke exposure was measured by an affirmative response by someone who smokes in the house. The poverty level was derived by NSCH based on the Department of Health and Human Services (DHHS)' guidelines in four levels of percent from the federal poverty level (FPL) (0–99; 100–199; 200–399; and 400 or above).

### 2.4. Data Analysis

All analyses were performed with the Statistical Package for the Social Sciences (SPSS) version 24 (IBM, New York, NY, USA) for complex samples. A sample plan was created in SPSS for complex samples where the stratum identifiers were STATE (state of residence) and SAMPLE (telephone sample type), and the primary sampling unit (PSU) variable was IDNUMR (unique household identifier). The sample weight (NSCHWT) added accounted for a representative U.S. sample of noninstitutionalized children and for nonresponse. Frequency, percent, and 95^th^ percent confidence intervals by cross tabulation are presented in Table 1, for risk factors of obesity. Tables 2 and 3 show the percent associations of sociodemographics with preventive medical care and adequate health insurance, respectively.  Table 4 presents the final complex logistic regression model testing the main effects of all the variables in the study and interactions by ethnicity, sex, and age categories. All main effects of obesity indicators and significant interactions were retained. Analysis for differences among race/ethnicity, neighborhood characteristics, and physical activity was conducted by cross tabulation and presented in the main text. Significance was considered at *p* < 0.05 for variables and *p* < 0.01 for models. Significance was based on the adjusted *F* and its degrees of freedom. The adjusted *F* is a variant of the second-order Rao–Scott-adjusted chi-square statistic.

## 3. Results

### 3.1. Obesity Risk Factors and Percent Overweight/Obese

Obesity risk factors for children with zBMI scores ≥ 85^th^ percentile (overweight and obese) are presented by cross tabulation (Table 1). Percent overweight/obese was significantly higher for children with the following characteristics: Hispanic and NHB as compared to NHW, youngest age category, male, no moderate-to-vigorous physical activity, high hours of television viewing (4 hours or more), inadequate sleep for age, second- and third-hand smoke in the house, perceived unsafe neighborhood, perceived nonsupportive neighborhood, and at or near the federal poverty level (FPL). Greater number of family meals per week was associated with overweight/obesity. Preventive medical care (*p*=0.159), insurance adequacy (*p*=0.113), inadequate sleep (*p*=0.062), use of computer and other electronic device (*p*=0.139), and having parks or playgrounds (*p*=0.133) were not significantly associated with overweight/obesity status.

### 3.2. Preventive Care and Health Insurance with Overweight/Obesity

Tables 2 and 3 show the percent (95^th^ % CI) of preventive medical care and health insurance adequacy levels by risk factors of overweight/obesity, respectively. Similar trends of significant indicators of overweight/obesity were found for no preventive medical care and inadequate or no health insurance in the past year for television viewing, physical activity, and family meals. Near or at the poverty level, watching television for four or more hours per day, and limited (1–3 days) or no moderate-to-vigorous physical activity were associated with no preventive medical care and inadequate or no health insurance. Eating family meals four to six nights per week was found for a higher percent of children with preventive medical care and adequate health insurance, whereas eating family meals every night was higher for children with no preventive medical care and inadequate or no health insurance. There were no differences for children eating family meals three or less times per week. Hispanic children had a higher percent with no preventive medical care and inadequate health insurance, and NHB children had a higher percent with inadequate or no health insurance as compared to NHW children. Neighborhood characteristics were poorer for children with inadequate or no health insurance, albeit only playground and parks in the neighborhood were the only significant neighborhood indicators of having preventive medical care. There was a slightly higher percent of boys as compared to girls with no preventive medical care. Inadequate sleep was associated with inadequate or no health insurance but not with preventive medical care.

### 3.3. Adjusted Model of Determinants of Overweight/Obesity

The final model for determinants of overweight/obesity is presented in Table 4. All interactions for sex, race/ethnicity, and age category were tested and significant interactions retained. Odds of overweight/obesity in the fully adjusted model were higher for males, Hispanics, non-Hispanic Black females, and children with high television viewing and low physical activity. The environmental factors that remained significant in the adjusted model were higher odds ratio of poverty, smoke exposure, and perceived unsupportive neighborhood. Medical care and insurance were no longer significant. Having family meals more often was associated with higher odds ratio of overweight/obesity.

### 3.4. Neighborhood Conditions and Physical Activity

Physical activity was significantly associated with each neighborhood characteristic. Parents reporting no parks or playgrounds in their community reported 12.5% (10.7, 14.5) of their children did not perform physical activity, whereas parents reporting having parks or playgrounds in their neighborhood reported 10.0% (9.2, 10.8) of their children did not perform physical activity. Parents reporting that their neighborhood was not supportive had 15.6% (13.7, 17.8) of their children not performing physical activity, while 9.4% (8.6, 10.2) did not perform physical activity in neighborhoods that parents reported as supportive. Similarly, parents who considered their neighborhoods unsafe had reported that 15.8% (12.9, 19.2) of their children did not perform physical activity versus 9.6% (9.0, 10.4) of children whose parents considered their neighborhood safe.

### 3.5. Physical Activity Race/Ethnicity

Physical activity was significantly different by race/ethnicity (*p* < 0.001). Hispanic children had the highest percent of “no moderate-to-vigorous physical activity,” 14.6% (12.2, 17.4) followed by NHB children, 12.8 (11.1, 14.7), other/multirace children, 10.3 (8.0, 13.1), and NHW children, 8.4 (7.8, 9.2). A significantly higher percent of NHW children, 32.9 (31.8, 34.1), participated 6-7 days per week in moderate-to-vigorous physical activity (at least 20 minutes each time) as compared to Hispanic children, 25.8 (22.8, 29.0), and NHB children, 28.4 (25.9, 31.0).

### 3.6. Neighborhood Conditions and Health Habits Race/Ethnicity

There were significant differences between race/ethnicity and neighborhood conditions: perceived safe neighborhood (*p* < 0.001), perceived supportive neighborhood (*p* < 0.001), parks and playgrounds (*p* < 0.001), and poverty level (*p* < 0.001) and health habits: smoke exposure in the house (*p* < 0.001), sleep (*p* < 0.001), family meals (*p* < 0.001), television viewing (*p* < 0.001), and use of electronic devices (*p* < 0.001). Over 20% of Hispanic and NHB parents report that their neighborhood is not safe as compared to 12% other/mixed-race parents and 6% NHW parents. Similarly, over 20% of Hispanic and NHB parents report that their neighborhood is not supportive as compared to 17% other/multirace parents and 10% NHW parents. Greater percent of NHW parents, approximately 19%, followed by 17% NHB parents report having no parks or playgrounds as compared to 12% of Hispanic parents and 15% other/multirace parents. Fewer NHW parents reported that their children received adequate sleep all nights of the week, 51.5% (50.2, 52.7) followed by other/mixed-race parents' report, 55.2% (52.4, 58.9), NHB parents' report, 57.5 (54.7, 60.3), and Hispanic parents' report, 61.3% (58.0, 64.6). Over one-third of NHB parents as compared to approximately 25% of other groups reported that they had family meals three times a week or less. Non-Hispanic Black children had the highest percent of watching television 4 or more hours per day, over one-quarter, followed by 14% of Hispanic children, 12% of other/mixed-race children, and 11% of NHW children. Parents of NHB children reported the highest percent of electronic device use for four hours or more, 25%, as compared to approximately 15% for other groups.

## 4. Discussion

Our results indicated that Hispanic and NHB children, ages 10–17 years, had 1.5 times higher percent of overweight/obesity as compared to Other/mixed-race and NHW children, which matches the trend for children, 2–19 years old [1]. We found a statistically higher percent of Hispanics, but not NHB, as compared to NHW children with no preventive medical care and inadequate health insurance. Our results suggest that racial/ethnic differences in resources are factors of obesity, which is in agreement with Caprio et al. [24]. In our study, a significantly higher percent of overweight/obese children exhibited the following behaviors: excessive television viewing and electronic device use and no or low-levels of participation in moderate-to-vigorous physical activity. Inactivity leads to weight gain, reduces aerobic fitness, and consequently makes physical activity more challenging [25].

We found that pubescent children had a higher percent of obesity compared to adolescents; yet, adolescents had a higher percent of most obesity risk factors including inadequate sleep, high sedentary behaviors, and low participation in moderate-to-vigorous physical activity. The physical activity level was found to decline for children from 10 to 19 years of age [26]. Obesity-related behaviors in adolescence are associated with adult obesity [27]. Physical activity declined in Black and White girls from baseline (ages 9 and 10 years) to follow-up (18 and 19 years) with 56% of Black girls and 31% of White girls reporting no physical activity outside of school requirements [28]. Sedentary behavior increases with age regardless of physical activity level for a cohort of children 8-9 years at baseline and 11-12 years at follow-up from northern England [29].

Greater number of family meals per week was associated with overweight/obesity and obesity in our study, while contrary results were found in the literature [12, 30]. Children whose family meals were of short duration and eaten in nondining areas were more likely to be obese in a sample of low-income, minority families [13]. Family meals data available for our study included one category for eating together, which could be at home or at a restaurant.

Environmental factors in our study that positively associated with overweight/obesity in children were smoke exposure, poverty, and neighborhood conditions (parents' perception of unsafe and unsupportive neighborhoods). Having parks or playgrounds in the community was not directly associated with overweight/obesity; however, a higher percent of children without these amenities did not perform any moderate-to-vigorous physical activity. Similarly, perceived lack of neighborhood support was associated with higher odds ratio of obesity and a lower percent of physical activity per week in the current study. Contradictory results were reported for South African children, where objective measures of neighborhood safety and socioeconomic status, not perceived neighborhood support, were associated with physical activity [31]. Neighborhood measures for barriers of physical activity may depend on the type of neighborhood. The built environment in a wealthy neighborhood could lead to healthier outdoor play, whereas a poor neighborhood could restrict play options for children [32]. Children, ages 10–12, who reported they had parks or playgrounds in their neighborhood had lower zBMI [32]. In our study, Hispanic children were more likely to report having parks or playgrounds in their neighborhood as compared to NHW children, yet NHW children had lower percentages of overweight and obesity compared to Hispanic children. Neighborhood wealth and race/ethnicity could confound parks and playgrounds as protective for risk of obesity. Inequalities of neighborhood facilities and support for physical activity may contribute to health disparities, such as childhood obesity [33]. The rise in childhood obesity across race/ethnicity may be due to the decline in safe environments and opportunities to participate in physical activity over the past decades in the U.S. and developing countries [27].

In our study, parents reporting having taken their child for preventive medical care or a wellness visit within the past 12 months was associated with the following: participation in physical activity; less time watching television and using electronic devices; and higher level above poverty. Hispanics had the highest percent reporting parks or playgrounds and the lowest percent of parents reporting that their child had primary medical care/wellness visit. Inadequate or no health insurance was highly correlated with no preventive medical care/wellness visit and other obesity indicators. According to the American Academy of Pediatrics' report, pediatricians should identify children at risk for obesity and work with families to improve dietary behaviors, reduce children's sedentary behaviors, and increase children's frequency and duration of moderate-to-vigorous physical activity to 60 minutes each day [4]. Preventive medical care should target issues specific to child's age, sex, and race/ethnicity and their parents' behavior and perceptions [4]. We found having preventive medical care to be associated with protective behaviors for obesity prevention. Health-care providers agree that a prescription for outdoor physical activity is essential to help combat obesity; however, prior to writing a prescription, assessment of the environment and potential strategies to increase physical activity outdoors need to be performed [34].

In the current study, child's behavioral factors that differed by race/ethnicity included sleep, sedentary behaviors, and physical activity. Lack of adequate sleep was apparent for a higher proportion of NHW children as compared to Hispanic children. Higher proportion of NHB children watched television (videos) or played video games and used electronic devices four hours or more as compared to other races/ethnicities. Limited participation in physical activity was more likely for minorities as compared to NHW. Parental responses that differed by race/ethnicity included providing preventive care, insurance adequacy, and the following perceptions: neighborhood safety, neighborhood support, and availability of playgrounds and/or parks. Hispanics had a higher percent of children with no or inadequate health insurance and without preventive medical care as compared to NHW. Compared to other groups, NHB children had fewer family meals together, on average, per week. Having family meals was more protective of obesity for Black as compared to White adolescents after a 10-year follow-up [30].

The association among parents' neighborhood perception, children's physical activity, and race/ethnicity have not been established. Budd et al. [35] reported racial/ethnic similarities in neighborhood perceptions and physical activity and that safe neighborhoods were associated with higher levels of physical activity for U.S. children. In our study, parents reporting that their children performing no physical activity reported higher percent of negative neighborhood characteristics: no parks or playgrounds, unsafe and unsupportive neighborhoods, and parents' perception of their neighborhood being supportive (independent and adjusted model) and safe (independently, only) were associated with lower rates of overweight/obesity (fully adjusted model). Singh et al. [9] recognized that U.S. children in the 2007 NSCH were less likely to be overweight or obese in neighborhoods with parks and playgrounds, albeit there was no significant relationship from 2011 to 2012 NSCH in our investigation. We established that Hispanic and NHB parents had a higher percent reporting having parks and playgrounds as compared to NHW parents. Reducing obesity risk, by providing an outlet for physical activity, could be confounded by a higher perception of the neighborhood as unsafe and unsupportive for Hispanic and NHB versus NHW parents.

We found that children with low physical activity and high television viewing were more likely to be overweight/obese as compared to under/normal weight in the fully adjusted model. Our results were congruent with several studies. Results of children from the National Health and Nutrition Surveys (NHANES), 2003–2006, indicated an association of children classified as obese as performing less moderate-to-vigorous physical activity; however, the associations depended on race/ethnicity [36]. High sedentary activity, such as television viewing over three hours, was shown to be associated with increased body fat in a cohort of children from preschool to early adolescence [37].

## 5. Limitations of the Study

This study had several limitations. Body mass index for sex and age categories were based on parents' report of their children's height and weight; however, body mass index for sex and age scores calculated using parent-reported height and measured weight did not differ significantly from scores using measured height and measured weight in children ages 2–17 from the U.S. [38]. The NHSC did not use the IOTF cut-off points when calculating zBMI estimates; however, the International Obesity Taskforce (IOTF) cut-off differences are small and do not impact the estimates of overweight or obesity prevalence [39]. Cases with missing values accounted for less than 1% of tested variables (except for income); however, they are eliminated and may have caused bias. Missing values for income were significant and not random causing the FPL to stray from representing the U.S. population. Data were available for parental perception of neighborhood conditions, but not actual neighborhood conditions. Despite the limitations, this study had several strengths including the use of a large dataset of a nationally representative sample of children, consideration of parental attitudes and behaviors, random selection of cell phone and of landlines, and the assessment of multifaceted environmental and behavioral relationship among childhood health factors.

## 6. Conclusion and Implication for Practice

Inadequate preventive medical care was related to several determinants of obesity in children. Children who did not have a wellness examination at least once, annually, had higher rates of physical inactivity, sedentary behaviors (television viewing and use of electronic devices), and exposure to second- and third-hand smoke, were more likely to be male and Hispanic.

Inadequate health insurance was positively related to inadequate preventive medical care. These results suggest that medical care plays a vital role in obesity prevention and that health-care providers and policy makers need to provide access and encouragement for wellness visits and adequate health care for children.

We found that perception of neighborhood support was associated with lower rates of overweight/obesity and higher frequency of moderate-to-vigorous physical activity. Community-based interventions promoting neighborhood parental cooperation may have potential to lower overweight and obesity in children. Future studies should continue to examine the potential for physical activity prescription by health-care professionals, the role of medical care, and parental cooperation as protective factors in childhood obesity.

## Figures and Tables

**Table 1:** Independent risk factors for overweight/obese versus normal weight children.

Variable	Overweight/obese: percent zBMI scores ≥ 85^th^ percentile (95^th^ CI)				*p*
Race/ethnicity	Hispanic	NHB	Other races	NHW	<0.001
	40.2^a^ (36.9, 43.6)	41.6^a^ (38.8, 44.4)	28.1^b^ (24.9, 31.4)	26.3^b^ (25.2, 27.4)	

Age category (years)	10–12	13–15	16‐17		<0.001
	37.7^a^ (36.0, 39.5)	29.9^b^ (28.2, 31.7)	24.4^c^ (22.5, 26.3)		

Sex	Boys		Girls		<0.001
	34.6 (33.2, 36.1)		27.8 (26.4, 29.3)		

Physical activity^#^	Days/week				<0.001
	0		11.9 (10.6, 13.3)		
	1–3		28.9 (27.1, 30.8)		
	4‐5		30.8 (29.1, 32.7)		
	6‐7		27.4 (25.6, 29.3)		

Television, video games	(Hours/day)				<0.001
	0 to <1		26.8^a^ (25.2, 28.4)		
	1 to <4		33.2^b^ (31.7, 34.8)		
	≥4		39.8^c^ (36.9, 42.8)		

Second- or third-hand smoke in house^‡^	Yes		42.7 (39.1, 46.3)		<0.001
	No		30.5 (29.4, 31.6)		

Perceived neighborhood safe	Yes		29.9 (28.9, 30.9)		<0.001
	No		42.1 (38.2, 46.1)		

Perceived neighborhood supportive	Yes		29.4 (28.3, 30.5)		<0.001
	No		42.2 (39.2, 45.3)		

Poverty level	(% FPL)				<0.001
	0–99		45.3^a^ (42.5, 48.1)		
	100–199		37.2^b^ (24.7, 39.8)		
	200–399		28.8^c^ (27.0, 30.5)		
	≥400		21.6^d^ (20.2, 23.1)		

zBMI = body mass index for age and sex; FPL = federal poverty level. *Note.* Data presented as percent within parameters. Parameters with the same letter were not significantly different. ^#^Times during the week that the child exercised, played a sport, or participated in physical activity for at least 20 minutes at the moderate-to-vigorous level. The following variables were not significantly different by zBMI: use of computer and other electronic devices (*p*=0.139); inadequate sleep (*p*=0.062); preventive medical care (*p*=0.159); health insurance adequacy (*p*=0.113); and parks or playgrounds (*p*=0.133). ^‡^Determined by affirmative response to “someone smokes in the house.”

**Table 2:** Preventive medical care and risk factors for obesity.

Parameter	No preventive medical care		Preventive medical care		*p*
	Percent (95^th^ CI)		Percent (95^th^ CI)		
Watch television (4 or more hr/day)	18.8 (16.6, 21.2)		13.1 (12.3, 13.9)		<0.001
Electronic devices (4 or more hr/day)	19.3 (17.1, 21.7)		15.0 (14.2, 15.9)		<0.001
Adequate sleep (3 or less nights/wk)	10.1 (8.5, 12.0)		10.7 (10.0, 11.5)		0.072
Physical activity moderate-to-vigorous (days/wk)					<0.001
0	24.8 (21.8, 28.1)		75.2 (71.9, 78.2)		
1–3	29.9 (28.2, 21.7)		80.1 (78.3, 81.8)		
4‐5	16.4 (14.9, 17.9)		83.6 (82.1, 85.1)		
6‐7	15.5 (14.0, 17.2)		84.5 (82.8, 86.0)		
Inadequate health insurance	39.4 (36.6, 42.2)		28.8 (27.7, 29.9)		<0.001
Second- or third-hand smoke exposure	7.2 (6.2, 8.4)		7.1 (6.6, 7.7)		0.894
% FPL (0–99)	25.0 (22.5, 27.7)		16.5 (15.6, 17.5)		<0.001
Family meals					0.001
0–3 times per wk.	27.7 (25.4, 30.1)		27.0 (26.0, 28.1)		
4–6 times per wk.	33.4 (30.8, 36.0)		38.7 (37.5, 39.9)		
Every night	38.9 (36.2, 41.8)		34.3 (33.2, 35.4)		
Sex	Boys	Girls	Boys	Girls	0.043
	54.0 (51.2, 56.8)	46.0 (43.2, 48.8)	50.9 (49.7, 52.1)	49.9 (47.9, 50.3)	
Parents perceived neighborhood not safe	13.0 (11.1, 15.2)		11.7 (10.8, 12.6)		0.226
Parents perceived no neighborhood support	15.5 (13.8, 17.4)		15.5 (14.6, 16.5)		0.968
Parents reported no playground and parks in neighborhood	19.6 (17.6, 21.9)		16.2 (15.4, 17.0)		0.002

% within race/ethnicity no preventive medical care	Hispanic	NHB	Other races	NHW	0.001
	21.8^a^ (19.1, 24.8)	17.8^b^ (15.8, 20.1)	19.1^a,b^ (16.3, 22.3)	16.7^b,c^ (15.7, 17.7)	

NHB = non-Hispanic Black; Other Race = race/ethnicity other than categories provided such as Asian or mixed race; NHW = non-Hispanic White; FPL = federal poverty level. *Note.* preventive medical care was considered affirmative if the parents reported their child had at least one wellness visit in the past 12 months. Physical activity was considered times during the week that the child exercised, played a sport, or participated in physical activity for at least 20 minutes that made (him/her) sweat and breathe hard? Values are percent (95^th^ CI) “within preventive care” unless otherwise specified. Age was not associated with preventive medical care (*p*=0.201).

**Table 3:** Health insurance adequacy and risk factors for obesity.

Parameter	Inadequate or no health insurance percent (95^th^ CI)		Adequate health insurance percent (95^th^ CI)		*p*
Watch television (4 or more hr/day)	15.7 (14.2, 17.4)		13.3 (12.5, 14.2)		0.007
Electronic devices (4 or more hr/day)	16.8 (15.2, 18.5)		15.4 (14.5, 16.3)		0.318
Adequate sleep (3 or less nights/wk)	12.5 (11.1, 13.9)		9.8 (9.1, 10.6)		<0.001
Physical activity moderate-to-vigorous (days/wk)					0.037
0	34.1 (30.6, 37.9)		65.9 (62.1, 69.4)		
1–3	32.0 (30.0, 34.1)		68.0 (65.9, 70.0)		
4‐5	29.5 (27.8, 31.3)		70.5 (68.7, 72.2)		
6‐7	29.6 (27.7, 31.4)		70.4 (68.6, 72.3)		
No preventive medical care	23.0 (21.1, 24.9)		15.7 (14.7, 16.7)		<0.001
Second- or third-hand smoke exposure	7.1 (6.2, 8.0)		7.2 (6.6, 7.8)		0.817
% FPL					<0.001
0–99	17.3 (15.6, 19.0)		18.5 (17. 5, 19.6)		
100–199	24.0 (22.1, 26.0)		19.8 (18.7, 20.9)		
Family meals (days per week)					0.002
0–3	27.7 (25.9, 29.6)		27.0 (25.9, 28.1)		
4–6	34.9 (32.2, 36,9)		38.9 (37.7, 40.2)		
Every night	37.4 (35.4, 39.5)		24.1 (32.9, 35.3)		
Sex	Boys	Girls	Boys	Girls	0.493
	31.0 (29.6, 32.5)	30.3 (28.8, 31.8)	69.0 (67.5, 70.4)	69.7 (68.2, 71.2)	
Parents perceived neighborhood not safe	15.5 (13.8, 17.4)		10.3 (9.4, 11.2)		<0.001
Parents perceived no neighborhood support	18.1 (16.5, 19.8)		14.4 (13.4, 15.4)		<0.001
Parents reported no playground and parks in neighborhood	18.6 (17.0, 20.2)		16.1 (15.2, 17.0)		0.006

% within race/ethnicity inadequate or no health insurance	Hispanic	NHB	Other races	NHW	<0.001
	37.1^a^ (33.9, 40.5)	28.2^b^ (25.7, 30.8)	34.3^a^ (30.7, 38.2)	28.5^b^ (27.4, 29.7)	

NHB = non-Hispanic Black; Other Race = race/ethnicity other than categories provided such as Asian or mixed race; NHW = non-Hispanic White; FPL = federal poverty level. *Note.* Age was not associated with health insurance adequacy (*p*=0.345).

**Table 4:** Odds ratio of children with zBMI scores ≥ 85^th^ percentile (overweight/obese) for key sociodemographic, behavioral, and parental factors.

Variable	Parameter	Odds (95^th^ CI)	*p*
Sex	Male	2.06 (1.64, 2.60)	<0.001
	Female (reference)	1.00	—

Race/ethnicity	—	—	<0.001
	Hispanic	1.49 (1.17, 1.90)	0.001
	NHB	1.91 (1.55, 2.37)	<0.001
	Other races	1.18 (0.92, 1.50)	0.191
	NHW (reference)	1.00	

Sex ∗ race/ethnicity	—	—	0.009
	Male, Hispanic	0.90 (0.65, 1.24)	0.530
	Male, NHB	0.63 (0.48, 0.83)	0.001
	Male, other races	0.77 (0.54, 1.11)	0.159
	NHW male and female all race/ethnicity (reference)	1.00	—

Age category (years)	—	—	<0.001
	10–12	2.41 (1.96, 2.95)	<0.001
	13–15	1.64 (1.33, 2.03)	<0.001
	16-17 (reference)	1.00	—

Gender ∗ age category	—	—	0.040
	Male, 10–12 years	0.74 (0.56, 0.97)	0.028
	Male, 13–15 years	0.71 (0.53, 0.94)	0.017
	Male 16-17 years (reference)	1.00	—

Family meals (times/week) on average	—	—	<0.001
	0–3	0.73 (0.64, 0.84)	<0.001
	4–6	0.83 (0.74, 0.94)	0.004
	Every night (reference)	1.00	—

^#^Television (hours/day)	—	—	0.001
	0–1	0.72 (0.61, 0.86)	<0.001
	>1 to <4	0.84 (0.72, 0.99)	0.040
	≥4 (reference)	1.00	—

Electronic devices	—	—	0.116
	0–1	0.98 (0.80, 1.12)	0.529
	>1 to <4	1.08 (0.92, 1.27)	0.361
	4 or more (reference)	1.00	

Adequate sleep	—	—	0.316
	3 nights or less	1.15 (0.96, 1.39)	0.133
	4–6 nights	1.03 (0.92, 1.16)	0.554
	Every night (reference)	1.00	—

Second- or third-hand smoke^±^	No	0.78 (0.65, 0.93)	0.005
	Yes (reference)	1.00	—

Physical activity (days/week)^‡^	—	—	<0.001
	0	1.38 (1.13, 1.69)	0.002
	1–3	1.41 (1.22, 1.62)	<0.001
	4–6	1.25 (1.09, 1.43)	0.001
	Every day (reference)	1.00	—

Poverty level (%FPL)	—	—	<0.001
	0–99	2.11 (1.79, 2.48)	<0.001
	100–199	1.68 (1.45, 1.96)	<0.001
	200–399	1.31 (1.16, 1.48)	<0.001
	400 and above (reference)	1.00	—

Supportive neighborhood	No	1.31 (1.12, 1.54)	0.001
	Yes (reference)	1.00	—

Safe neighborhood	No	1.10 (0.91, 1.34)	0.326
	Yes (reference)	1.00	—

Parks or playgrounds	No	1.02 (0.90, 1.15)	0.815
	Yes (reference)	1.00	

Preventive medical care	No	0.94 (0.82, 1.08)	0.400
	Yes (1 year or more) (reference)	1.00	—

Insurance adequacy	Adequate	0.96 (0.86, 1.07)	0.466
	Inadequate (reference)	1.00	—

zBMI = body mass index for age/sex; NHB = non-Hispanic Black; Other Race: Asian, Pacific Islander, Native American, or mix races other than Hispanic; FPL = federal poverty level. *Note.* Analysis presented is the final complex logistic regression model with adequate model fit (*p* < 0.01). ^#^*Television* was time spent watching television and videos or playing video games (on average per weekday). ^±^*Smoke exposure* was determined by affirmative response to “someone smokes in the house.” ^‡^*Physical activity* was considered times during the week that the child exercised, played a sport, or participateed in physical activity for at least 20 minutes that made (him/her) sweat and breathe hard? *Supportive neighborhood* was perceived by parents and based on NSCH derived from questions concerning cooperation and trust.

## Data Availability

Data are publicly available [19].
